# Prognostic Value of Endothelial Progenitor Cells in Acute Myocardial Infarction Patients

**DOI:** 10.1155/2023/4450772

**Published:** 2023-09-28

**Authors:** Gongjie Ye, Xiaodan Chen, Yinchao Zhou, Jianqing Zhou, Yongfei Song, Xiaoyong Yang, Lei Yang

**Affiliations:** ^1^Department of Intensive Care Unit, Ningbo Medical Centre Lihuili Hospital, Ningbo University, Ningbo, Zhejiang 315040, China; ^2^Department of Clinical Laboratory, Ningbo Medical Centre Lihuili Hospital, Ningbo University, Ningbo, China; ^3^Ningbo University, Ningbo 315211, Zhejiang, China; ^4^Internal Medicine-Cardiovascular Department, Ningbo Medical Centre Lihuili Hospital, Ningbo University, Ningbo, China; ^5^Ningbo Institute for Medicine and Biomedical Engineering Combined Innovation, Ningbo Medical Centre Lihuili Hospital, Ningbo University, Ningbo 315000, Zhejiang, China; ^6^Department of Rehabilitation, Zhenhai Longsai Hospital, Ningbo 315000, Zhejiang, China; ^7^Department of Intensive Care Unit, Zhenhai Longsai Hospital, 6 Gulou West Road, Chengguan, Zhaobaoshan Street, Zhenhai District, Ningbo 315299, Zhejiang, China

## Abstract

**Objective:**

To determine prognostic role of endothelial progenitor cells (EPCs) in intensive care patients with acute myocardial infarction (AMI).

**Materials and Methods:**

From December 2018 to July 2021, a total of 91 eligible patients with AMI were consecutively examined in a single intensive care unit (ICU) in China. Patients with a history of acute coronary artery disease were excluded from the study. Samples were collected within 24 hr of onset of symptoms. EPCs, defined as coexpression of CD34+/CD133+ cells or CD133+/CD34+/KDR+, were studied using flow cytometry and categorized by quartiles. Based on the 28-days mortality outcome, the patients were further divided into two groups: death and survival. The study incorporated various variables, including cardiovascular risk factors such as body mass index, hypertension, diabetes, hypercholesterolemia, atherosclerotic burden, and medication history, as well as clinical characteristics such as APACHEⅡscore, central venous-arterial carbon dioxide difference (GAP), homocysteine, creatinine, C-reactive protein, HbAlc, and cardiac index. Cox regression analysis was employed to conduct a multivariate analysis.

**Results:**

A total of 91 patients with AMI who were admitted to the ICU were deemed eligible for inclusion in the study. Among these patients, 23 (25.3%) died from various causes during the follow-up period. The counts of EPCs were found to be significantly higher in the survival group compared to the death group (*P* < 0.05). In the univariate analysis, it was observed that the 28-days mortality rate was associated with the several factors, including the APACHEⅡscore (*P*=0.00), vasoactive inotropic score (*P*=0.03), GAP (*P*=0.00), HCY (*P*=0.00), creatinine (*P*=0.00), C-reactive protein (*P*=0.00), HbAlc (*P*=0.00), CI (*P*=0.01), quartiles of CD34+/CD133+ cells (*P*=0.00), and quartiles of CD34+/CD133+/KDR+ cells (*P*=0.00). CD34+/CD133+/KDR+ cells retained statistical significance in Cox regression models even after controlling for clinical variables (HR: 6.258 × 10^−10^ and *P*=0.001). Nevertheless, no significant correlation was observed between CD34+/CD133+ cells and all-cause mortality.

**Conclusions:**

The decreased EPCs levels, especially for CD34+/CD133+/KDR+ cells subsets, were an independent risk factor for 28-days mortality in AMI patients.

## 1. Introduction

Acute myocardial infarction (AMI), a prime example of endothelial injury, is further compounded by the occurrence of superimposed thrombosis [1]. Subsequent to endothelial damage resulting from a heart attack, endothelial progenitor cells (EPCs) are released from the bone marrow and play a role in the development of neovascularization in adults [2]. The levels of EPCs have been linked to cardiovascular disease, with their modulation believed to be influenced by the presence of vascular risk factors and the efficacy of medication in managing these risk factors [3–7]. The association between elevated levels of EPCs and the probability of future cardiovascular events in individuals diagnosed with stable coronary artery disease has been established [8, 9].

There is increasing evidence indicating a correlation between EPCs levels and the outcomes of patients with AMI [10]. However, due to significant heterogeneity among studies, the precise role of EPCs in predicting prognosis in AMI remains uncertain [11–17]. Consequently, our objective was to assess the relationship between EPCs levels and the outcome of AMI.

## 2. Materials and Methods

### 2.1. Ethics Statement

A prospective trial was conducted at a tertiary hospital in China, adhering to the principles outlined in the Declaration of Helsinki. The study protocol received approval from the Ethics Review Board for Clinical Studies of Ningbo Medical Centre Lihuili Hospital (Approval Number: KY2019PJ044). Informed consent was obtained from all patients, their legal representatives, and agents involved in the study.

### 2.2. Study Population

The diagnosis of AMI involved the presence of self-reported chest pain, accompanied by evidence of ischemia on a 12-lead electrocardiogram (EKG) or significantly elevated levels of cardiac enzymes exceedingly twice the upper limit of normal as observed in angiography. Based on the EKG findings, patients with AMI were categorized into two subtypes: ST-elevation myocardial infarctions (STEMIs) or non-ST-elevation myocardial infarctions (NSTEMIs). STEMI was defined as ST elevation of 0.1 mV or more in at least two contiguous leads, while NSTEMI required the presence of ischemic changes, such as ST-segment deviation or T-wave inversion. Between December 2018 and June 2021, the intensive care unit (ICU) of Ningbo Medical Centre Lihuili Hospital admitted patients diagnosed with STEMI and NSTEMI. Consistent with prevailing guidelines, all patients underwent appropriate therapeutic interventions [18–21].

The study employed specific inclusion and exclusion criteria. Inclusion criteria encompassed the diagnostic criteria for AMI as defined by the American College of Cardiology (ACC), American Heart Association (AHA), and the European Heart Association (ECH) [18–21]. Additionally, patients admitted to ICU within 24 hr and aged 16 years or older were included. Conversely, exclusion criteria consisted of patients with previously documented acute coronary artery disease, donors, those using statins, angiotensin-converting enzyme inhibitors, activated protein C, or experiencing hemorrhagic shock, chronic obstructive pulmonary disease, hydrocortisone, or isolated acute respiratory distress syndrome.

### 2.3. Blood Sampling and Isolation of Peripheral Blood Mononuclear Cells (PBMCs)

Twenty milliliters of blood were collected from either the central venous catheter or peripheral veins within 24 hr of symptom onset and processed within 1 hr. PBMCs were isolated from the peripheral blood using Ficoll gradient centrifugation. The Ficoll solution (Tianjin Haoyang Biological Products, China) was gently poured over the peripheral blood diluted 1 : 1 in phosphate-buffered saline (PBS). Initially, the cells in the interphase were centrifuged at room temperature for 25 min at 700 G, followed by aspiration and centrifugation at 300 G for 10 min. Following the removal of the supernatant, the pellet underwent an incubation period of 8 min with erythrocyte lysis buffer. Furthermore, the cells were subjected to two washes with PBS, followed by centrifugation at 300 G for 8 min, and subsequently analyzed using flow cytometry.

### 2.4. Flow Cytometry

We employed a double- or three-color immunofluorescence staining technique to assess the expression of cell-surface antigens on EPCs. PBMCs, consisting of 1 million cells, were incubated at a temperature of 4°C for a duration of 30 min with 5 *μ*L of PE-conjugated anti-human vascular endothelial growth factor (VEGFR-2) (BioLegend, USA), FITC-conjugated anti-human CD34 (BioLegend, USA), and APC-conjugated anti-human CD133 (BioLegend, USA). Subsequently, the cells were subjected to three washes at 300 G for 5 min and then resuspended in 500 *μ*L of PBS. Flow cytometry analysis was conducted using a FACS Calibur flow cytometer (BD Biosciences, USA) and FlowJo version 10.4 software (FlowJo, LLC, Ashland, OR, USA). The EPCs counts were quantified as percentages relative to the total PBMCs in each participant of the study.

### 2.5. Variables Analyzed

All patients were assessed for the following traditional cardiovascular risk factors: age, sex, body mass index (BMI), hypertension, diabetes, hypercholesterolemia, atherosclerotic burden (AB), and previous medication use. Additionally, data on demographics, clinical variables, plasma biomarkers, medical history, medication use, and behavior were collected. Plasma biomarkers consisted of cardiac troponin I (cTnI) and N-terminal pro-brain natriuretic peptide (NT-proBNP). Vasoactive inotropic score (VIS) and central venous-arterial carbon dioxide difference (P(cv-a) CO2, GAP) were also calculated for each patient. The primary outcome measure for patients diagnosed with AMI was all-cause mortality, assessed after a period of 28 days from admission.

### 2.6. Data Analysis

Statistical analyses were performed using SPSS version 25.0 (SPSS Inc., Chicago, IL). Kolmogorov–Smirnov tests were utilized to assess the presence of normal and non-Gaussian distributions. Both nonparametric and parametric methods were employed. Multivariate logistic regression analysis was conducted to identify the independent factors influencing the prognosis of patients with AMI, and statistically significant variables from the univariate analysis were included as independent variables in the multivariate analysis. The significance level for the examination was set at *α* = 0.05, and a *P*-value of less than 0.05 was considered statistically significant.

## 3. Results

### 3.1. Study Population

Between December 2018 and June 2021, a cohort of 186 patients diagnosed with AMI were admitted to ICU. Ultimately, a total of 91 AMI patients were included in the final analysis, with 69 experiencing STEMI and 22 experiencing NSTEMI. All patients underwent coronary angiography, with the exception of AB distribution, no significant differences were observed between the baseline characteristics or clinical variables of STEMI and NSTEMI patients. The demographic and clinical characteristics can be found in Table S1 and S2.

EPCs (CD34+/CD133+ cells) were partitioned equally into four quartiles, namely Q1 (0–0.288%), Q2 (0.288%–0.425%), Q3 (0.430%–0.555%), and Q4 (>0.555%), based on their relative numbers. Similarly, the EPCs (CD34+/CD133+/KDR+ cells) were also categorized into quartiles: Q1 (0–0.129%), Q2 (0.129%–0.170%), Q3 (0.170%–0.230%), and Q4 (>0.230%). The APACHE II score, GAP, homocysteine (HCY), creatinine, C-reactive protein, HbAlc, cardiac index (CI), and AB exhibited significant variations among the four groups of EPCs (CD34+/CD133+ cells). Additionally, a negative correlation was observed between basal EPCs (CD34+/CD133+ cells) levels and the following parameters: APACHE II score (*P*=0.00), GAP (*P*=0.04), HCY (*P*=0.01), creatinine (*P*=0.01), C-reactive protein (*P*=0.04), HbAlc (*P*=0.04), AB (*P*=0.01), and CI (*P*=0.01). Regarding EPCs (CD34+/CD133+/KDR + cells), there was a significant association between lower basal EPCs (CD34+/CD133+/KDR + cells) levels and higher APACHEⅡscore (*P*=0.00), higher creatinine (*P*=0.03), higher HbAlc (*P*=0.04), higher Killip classifications (*P*=0.04), higher AB (*P*=0.02), and a lower CI (*P*=0.03). A comprehensive overview of the univariate analysis can be found in Tables 1 and 2.

### 3.2. Incidence of Outcomes of AMI Patients

Among a cohort of 91 patients who presented with AMI, a total of 23 individuals (25.3%) experienced mortality from various causes during a 28-days follow-up period. This group, referred to as the “death group,” comprised 16 deaths that transpired within the initial 7 days of hospital admission, while the remaining seven deaths occurred between the 7th and 28th days of admission. The remaining 68 patients (74.7%) successfully survived the aforementioned time frame, as the “survival group”.

A statistically significant increases EPCs counts CD34+/CD133+ cells quartiles (*P*=0.00), and CD34+/CD133+/KDR+ cells were observed in the survival group compared with the death group (*P* < 0.05). Univariate analysis revealed that 28-days mortality was associated with the several factors, including APACHEⅡscore (*P*=0.00), VIS (*P*=0.03), GAP (*P*=0.00), HCY (*P*=0.00), creatinine (*P*=0.00), C-reactive protein (*P*=0.00), HbAlc (*P*=0.00), CI (*P*=0.01), CD34+/CD133+ cells quartiles (*P*=0.00), and CD34+/CD133+/KDR+ cells quartiles (*P*=0.00). The detailed analysis can be found in Table 3.

**Figure 1:**
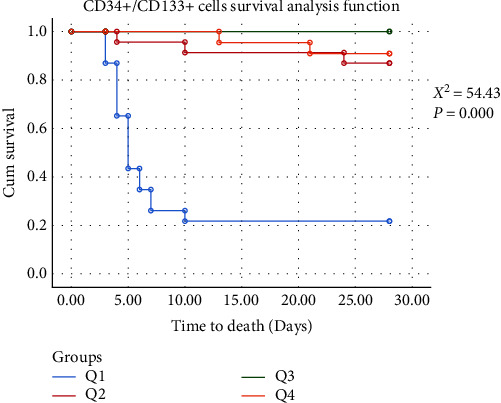
Kaplan–Meier survival curve according to CD34+/CD133+ cells (%) quartiles.

**Figure 2:**
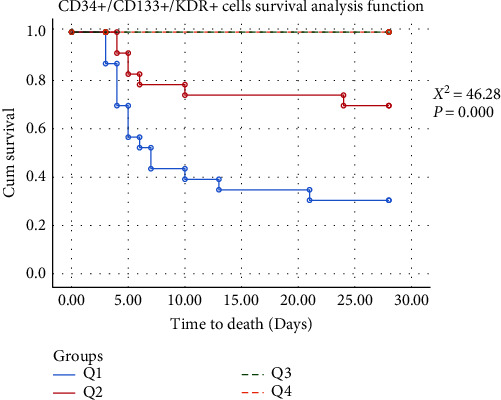
Kaplan–Meier survival curve according to CD34+/CD133+/KDR+ cells (%) quartiles.

To explore potential correlations between survival trajectory and quartiles of EPCs, we categorized 28-days mortality into three subgroups: the survival group, the death group within 7 days, and the death group from the 7th to 28th days. Notably, both quartiles of CD34+/CD133+ cells and CD34+/CD133+/KDR+ cells exhibited significant differences across these three groups (Figures 1 and 2). Moreover, the survival group exhibited the highest EPCs count.

In Cox regression models (also known as the proportional hazards model, is a semiparametric regression model), the significance of CD34+/CD133+/KDR+ cells persisted even after controlling for various clinical variables including age, gender, BMI, APACHEⅡscore, ST-AMI, VIS, GAP, HCY, creatinine, C-reactive protein, HbAlc, and CI (HR: 6.258 × 10^−10^ and *P* < 0.01). However, no significant association was observed between CD34+/CD133+ cells and all-cause mortality (*P* > 0.05), as indicated in Table 4.

## 4. Discussion

In accordance with our research, a lower count of EPCs exhibited a correlation with increased mortality within 28 days among individuals diagnosed with AMI. These findings are consistent with the previous studies conducted on AMI patients, which have yielded comparable outcomes. Based on the findings of the procell study, it has been determined that basal EPCs levels possess the ability to forecast forthcoming vascular events in a cohort of 100 patients diagnosed with AMI within the initial 6 months of posttreatment monitoring. Through the utilization of a multivariate Cox regression analysis, it has been established that there exists an independent correlation between EPCs counts within the lowest quartile (HR: 10.33 and *P*=0.032) and the occurrence of new vascular events, encompassing new acute coronary syndrome, transient ischemic attack, stroke, or any hospitalization or death resulting from the cardiovascular causes [11]. In a separate investigation involving 529 individuals diagnosed with acute coronary syndrome, it was found that subjects with low levels of EPCs exhibited a 2.46-fold increase in the likelihood of all-cause mortality (95% CI 1.18–51.3) [22].

In this study, our inclusion criteria were limited to patients diagnosed with AMI, due to their critical condition and elevated mortality risk. Consequently, our research concentrated on individuals who were admitted to the hospital within 24hr of the initial event, and we monitored them for a brief duration subsequent to their admission. It is imperative to acknowledge that our study's focus solely on AMI patients admitted to our ICU may result in the exclusion of numerous AMI patients who did not require ICU treatment due to their less severe condition. Consequently, this limitation may pose challenges when attempting to extrapolate our findings to a broader population.

AMI leads to a time-dependent increase in the mobilization of EPCs from the bone marrow to the peripheral circulation [14, 17]. This mobilization process initiates shortly after an AMI, reaches its peak after several days, and returns to baseline levels within 60 days [23]. The occurrence of myocellular necrosis triggers an acute inflammatory response, resulting in the upregulation of hypoxia-inducible factor 1-alpha (HIF-1*α*). Consequently, the expression of stromal cell-derived factor-1 Alpha (SDF-1*α*) is stimulated, acting as a chemotactic signal for the recruitment of EPCs to ischemic tissues [24, 25].

In our study, we did not investigate the potential correlation between peripheral and bone marrow progenitor cells in patients with AMI. Despite the aforementioned findings, it remains uncertain whether the reduced levels of EPCs observed in AMI patients are a result of diminished progenitor cell reserves in the bone marrow or impaired mobilization of progenitor cells from the bone marrow [26–28]. Moreover, although the mechanism by which EPCs repair the endothelium remains incompletely understood [29–31], the application of stem cell therapy for vascular system diseases has demonstrated encouraging outcomes. However, it is imperative to conduct larger-scale trials to assess the therapeutic effectiveness and identify the most suitable patient population.

Currently, two distinct strategies may be employed for the application of EPCs-based cellular therapy, particularly in the treatment of AMI, acute cerebral infarction, limb ischemia, and similar conditions [32]. One therapeutic strategy entails the administration of stem cells or endothelial progenitors via local injection directly into the ischemic tissue. Another viable approach to substitute exogenous administration is the endogenous stimulation of EPCs [33, 34]. In the presence of ischemia, the expression of VEGF, stromal cell-derived factor 1 (SDF-1), hepatocyte growth factor (HGF), and endothelin 1 (ET-1) is increased, thereby stimulating the recruitment of EPCs to the ischemic sites [32]. Furthermore, the identical factors that facilitate the mobilization of EPCs, such as VEGF and SDF-1, alongside various pharmaceutical agents including statins and erythropoietin (EPO), significantly contribute to the migration, viability, and specialization of EPCs [35–37].

In the future study, our focus was on EPCs obtained from individuals diagnosed with AMI. These cells will be divided into two distinct groups: the experimental group, which will be cultured in the endothelial cell growth medium-2 containing factors such as VEGFR, SDF-1, ET-1 etc.; and the control group, which will be cultured in the regular fetal bovine serum medium. In this study, we aim to assess the directional differentiation, proliferation, and migration capacity of two different states of EPCs. The objective is to establish a solid groundwork for subsequent drug investigations targeting AMI.

## 5. Conclusions

Our study demonstrates that lower EPC levels, especially for CD34+/CD133+/KDR+ cells subsets, were associated with higher mortality in the patients with AMI. These results contribute to the advancement of knowledge regarding the inherent regenerative responses following AMI and are expected to inspire the formulation of innovative approaches for cell-based therapies.

## Figures and Tables

**Table 1:** Comparison of study variables among EPCs (CD34+/CD133+cells) quartiles (M (Q25 and Q75) or M ± SD).

	Q1 *N* = 23 (0–0.288%)	Q2 *N* = 23 (0.288%–0.425%)	Q3 *N* = 23 (0.430%–0.555%)	Q4 *N* = 22 (>0.555%)	*t*/*F*/*x*^*2*^	*P*
Male (*n* (%))	17 (73.9)	16 (69.6)	18 (78.3)	18 (81.8)	1.04	0.79
Age (years)	70.3 ± 11.7	67.3 ± 14.4	64.7 ± 15.2	60.9 ± 17.2	1.67	0.17
BMI (kg/m^2^)	22.5 ± 2.4	22.3 ± 2.5	22.1 ± 2.1	21.9 ± 2.4	0.34	0.80
APACHEⅡscore (points)	25.8 ± 7.9	18.2 ± 6.8	14.7 ± 3.8	15.8 ± 5.6	14.67	0.00
STEMI (*n* (%))	17 (73.9)	15 (65.2)	19 (82.6)	18 (86.4)	3.42	0.33
cTnI (*μ*g/L)	66.8 (42.4, 85.2)	48.5 (38.6, 76.6)	48.5 (37.1, 79.3)	43.5 (32.8, 75.5)	2.76	0.43
NT-proBNP (ng/L)	9920.9 (6483.4, 19339.0)	9999.4 (8659.3, 14098.3)	8882.9 (7208.2, 13822.9)	7824.7 (5870.9, 13802.9)	2.33	0.51
VIS (points)	15.7 ± 3.0	15.0 ± 3.1	14.9 ± 2.6	15.3 ± 3.3	0.30	0.82
GAP (mm Hg)	6.1 ± 1.4	5.5 ± 1.0	5.3 ± 0.7	5.4 ± 0.7	2.95	0.04
HCY (*μ*mol/L)	29.4 (19.2, 58.6)	17.9 (11.9, 30.8)	21.6 (15.9, 37.4)	16.4 (10.2, 29.5)	10.7	0.01
Creatinine (*μ*mol/L)	95.8 (83.3, 119.2)	69.5 (60.5, 85.2)	88.6 (65.4, 98.3)	73.8 (59.7, 98.6)	11.09	0.01
C-reactive protein (mg/L)	43.4 (20.3, 60.0)	21.2 (4.9, 44.5)	13.9 (7.5, 36.6)	17.4 (8.6, 43.7)	8.19	0.04
HbAlc (%)	6.6 ± 2.0	5.9 ± 1.5	6.3 ± 1.8	5.2 ± 0.8	2.99	0.04
CI (L/min/m^2^)	2.2 ± 0.8	2.8 ± 0.7	2.6 ± 0.7	2.3 ± 0.5	4.19	0.01
Killip classifications (n (%))					5.74	0.12
Level I–II	3 (13.0)	10 (43.5)	9 (39.1)	7 (31.8)		
Level III–IV	20 (87.0)	13 (56.5)	14 (60.9)	15 (68.2)		
AB (*n* (%))					2.49	0.01
Three territories	7 (30.4)	5 (21.7)	3 (13.0)	2 (9.1)		
Two territories	8 (30.8)	5 (21.7)	1 (4.3)	5 (22.7)		
One territory	8 (30.8)	13 (56.5)	19 (82.6)	15 (68.2)		

AMI, indicates acute myocardial infarction; BMI, body mass index; STEMI, ST-elevation MI; VIS, vasoactive inotropic score; GAP, central venous-arterial carbon dioxide difference; HCY, homocysteine; CI, cardiac index; AB, atherosclerotic burden.

**Table 2:** Comparison of study variables among EPCs (CD34+/CD133+/KDR+ cells) quartiles (M (Q25 and Q75) or M ± SD).

	cEPCs Q1 *N* = 23 (0–0.129%)	cEPCs Q2 *N* = 23 (0.129%–0.170%)	cEPCs Q3 *N* = 23 (0.170%–0.230%)	cEPCs Q4 *N* = 22 (>0.230%)	*t*/*F*/*x*^*2*^	*P*
Male (*n* (%))	16 (69.6)	15 (65.2)	21 (91.3)	17 (77.3)	4.94	0.18
Age (years)	68.3 ± 14.2	69.7 ± 12.1	62.3 ± 14.4	63.0 ± 17.9	1.46	0.23
BMI (kg/m^2^)	22.6 ± 2.5	22.2 ± 2.4	21.4 ± 1.9	22.6 ± 2.5	1.19	0.32
APACHEⅡscore (points)	24.0 ± 8.8	18.9 ± 7.2	15.9 ± 4.8	15.6 ± 5.9	7.33	0.000
STEMI (*n* (%))	19 (82.6)	15 (65.2)	18 (78.3)	17 (77.3)	2.09	0.55
cTnI (*μ*g/L)	54.4 (38.6, 80.2)	75.1 (42.4, 80.2)	58.9 (37.0, 76.6)	42.8 (32.8, 62.9)	5.47	0.14
NT-proBNP (ng/L)	8882.9 (6029.1, 19098.3)	13822.9 (8659.3, 18100.7)	8949.4 (6823.8, 13822.9)	8916.2 (6371.6, 13156.9)	4.84	0.18
VIS (points)	16.3 ± 2.9	14.5 ± 3.4	15.0 ± 2.9	15.0 ± 2.6	1.45	0.23
GAP (mm Hg)	5.9 ± 1.5	5.7 ± 0.9	5.5 ± 0.8	5.0 ± 0.7	1.69	0.18
HCY (*μ*mol/L)	27.5 (16.4, 49.3)	29.1 (20.3, 34.2)	17.7 (10.8, 24.3)	15.0 (11.3, 30.9)	11.76	0.008
Creatinine (*μ*mol/L)	92.2 (74.3, 121.6)	84.5 (70.2, 91.0)	65.5 (58.7, 88.6)	76.5 (59.9, 102.3)	8.73	0.03
C-reactive protein (mg/L)	40.5 (10.6, 49.3)	21.2 (9.9, 60.0)	12.8 (6.9, 30.4)	23.4 (9.8, 53.9)	6.02	0.11
HbAlc (%)	6.6 ± 1.8	5.7 ± 1.5	5.6 ± 1.4	6.1 ± 1.8	1.73	0.17
CI (L/min/m^2^)	2.2 ± 0.7	2.3 ± 0.6	2.7 ± 0.7	2.6 ± 0.6	3.18	0.03
Killip classifications (*n* (%))					8.24	0.04
Level I–II	4 (17.4)	10 (43.5)	11 (47.8)	4 (18.2)		
Level III–IV	19 (82.6)	13 (56.5)	12 (52.2)	18 (81.8)		
AB (*n* (%))					2.40	0.02
Three territories	7 (30.4)	7 (30.4)	3 (13.0)	0 (0.0)		
Two territories	8 (30.8)	2 (8.7)	6 (26.1)	3 (13.6)		
One territory	8 (30.8)	14 (60.9)	14 (60.9)	19 (86.4)		

AMI, indicates acute myocardial infarction; BMI, body mass index; STEMI, ST-elevation MI; VIS, vasoactive inotropic score; GAP, central venous-arterial carbon dioxide difference; HCY, homocysteine; CI, cardiac index; AB, atherosclerotic burden.

**Table 3:** Comparison of study variables between survival group and death group (M (Q25 and Q75) or M ± SD).

	Survival group (*N* = 68)	Death group (*N* = 23)	*t*/*U*/*x*^*2*^	*P*
Male (*n* (%))	52 (76.5)	17 (73.9)	0.06	0.80
Age (years)	65.1 ± 15.3	68.1 ± 13.5	0.84	0.41
BMI (kg/m^2^)	22.2 ± 2.4	22.1 ± 2.3	0.20	0.84
APACHEⅡscore (points)	15.5 ± 5.0	28.0 ± 5.8	9.94	0.00
STEMI (*n* (%))	52 (76.5)	17 (73.9)	0.06	0.80
cTnI (*μ*g/L)	47.8 (37.1, 76.6)	75.1 (47.0, 129.0)	2.20	0.03
NT-proBNP (ng/L)	8949.4 (6950.2, 13822.9)	13822.9 (6483.4, 19339.0)	1.41	0.16
VIS (points)	14.8 ± 2.9	16.4 ± 2.9	2.24	0.03
GAP (mm Hg)	5.3 ± 0.8	6.2 ± 1.3	3.94	0.00
HCY (*μ*mol/L)	17.8 (12.0, 30.2)	40.3 (22.2, 58.6)	4.41	0.00
Creatinine (*μ*mol/L)	72.9 (60.1, 91.7)	95.8 (88.4, 119.2)	4.35	0.00
C-reactive protein (mg/L)	14.3 (6.7, 35.2)	49.3 (40.5, 68.9)	5.17	0.00
HbAlc (%)	5.7 ± 1.5	6.9 ± 1.9	3.25	0.00
CI (L/min/m^2^)	2.6 ± 0.6	2.2 ± 0.8	2.53	0.01
CD34+/CD133+ cells (%)	0.50 ± 0.17	0.37 ± 0.18	19.56	0.00
CD34+/CD133+/KDR+ cells (%)	0.19 ± 0.06	0.14 ± 0.02	28.96	0.00
CD34+/CD133+ cells quartiles (*n* (%))			49.77	0.00
Q1	5 (21.7)	18 (78.3)		
Q2	21 (91.3)	2 (8.7)		
Q3	23 (100.0)	0 (0.0)		
Q4	20 (90.9)	2 (9.1)		
CD34+/CD133+/KDR+ cells quartiles (*n* (%))			44.04	0.00
Q1	6 (26.1)	17 (73.9)		
Q2	17 (73.9)	6 (26.1)		
Q3	23 (100.0)	0 (0.0)		
Q4	22 (100.0)	0 (0.0)		

AMI, indicates acute myocardial infarction; STEMI, ST-elevation MI; BMI, body mass index; VIS, vasoactive inotropic score; GAP, central venous-arterial carbon dioxide difference; HCY, homocysteine; CI, cardiac index; AB, atherosclerotic burden.

**Table 4:** Association between EPCs counts and all-cause death in AMI patients.

	*β*	*S*_*¯x*_	Wald	*P* value	HR	HR (95% CI)
CD34+/CD133+ cells	−4.677	2.425	3.721	0.054	0.009	8.00 × 10^−4^–1.078
CD34+/CD133+/KDR+ cells	−21.192	6.270	11.423	0.001	6.258 × 10^−10^	2.878 × 10^−15^–1.36^*∗*^10^−4^

## Data Availability

Data supporting the findings of this study can be obtained from the corresponding author upon request.

## Supplementary Materials

Supplementary Materials Table S1: baseline characteristics among subjects with AMI. Table S2: baseline clinical variables among subjects with AMI (M (Q25 and Q75) or M ± SD).

## References

[1] Lerman A., Holmes D. R., Herrmann J., Gersh B. J. (2007). Microcirculatory dysfunction in ST-elevation myocardial infarction: cause, consequence, or both?. *European Heart Journal*.

[2] Rehman J., Li J., Orschell C. M., March K. L. (2003). Peripheral blood “Endothelial Progenitor Cells” are derived from monocyte/macrophages and secrete angiogenic growth factors. *Circulation*.

[3] Fadini G. P., Agostini C., Sartore S., Avogaro A. (2007). Endothelial progenitor cells in the natural history of atherosclerosis. *Atherosclerosis*.

[4] Umemura T., Soga J., Hidaka T. (2008). Aging and hypertension are independent risk factors for reduced number of circulating endothelial progenitor cells. *American Journal of Hypertension*.

[5] Vasa M., Fichtlscherer S., Adler K. (2001). Increase in circulating endothelial progenitor cells by statin therapy in patients with stable coronary artery disease. *Circulation*.

[6] Hill J. M., Zalos G., Halcox J. P. J. (2003). Circulating endothelial progenitor cells, vascular function, and cardiovascular risk. *New England Journal of Medicine*.

[7] Vasa M., Fichtlscherer S., Aicher A. (2001). Number and migratory activity of circulating endothelial progenitor cells inversely correlate with risk factors for coronary artery disease. *Circulation Research*.

[8] Schmidt-Lucke C., Roössig L., Fichtlscherer S. (2005). Reduced number of circulating endothelial progenitor cells predicts future cardiovascular events: proof of concept for the clinical importance of endogenous vascular repair. *Circulation*.

[9] Werner N., Kosiol S., Schiegl T. (2005). Circulating endothelial progenitor cells and cardiovascular outcomes. *New England Journal of Medicine*.

[10] Rigato M., Avogaro A., Fadini G. P. (2016). Levels of circulating progenitor cells, cardiovascular outcomes and death: a meta-analysis of prospective observational studies. *Circulation Research*.

[11] Cuadrado-Godia E., Regueiro A., Núñez J. (2015). Endothelial progenitor cells predict cardiovascular events after atherothrombotic stroke and acute myocardial infarction. a PROCELL substudy. *PLOS ONE*.

[12] Padfield G. J., Tura-Ceide O., Freyer E. (2013). Endothelial progenitor cells, atheroma burden and clinical outcome in patients with coronary artery disease. *Heart*.

[13] Yu C. W., Choi S.-C., Hong S. J. (2013). Cardiovascular event rates in patients with ST-elevation myocardial infarction were lower with early increases in mobilization of Oct4highNanoghigh stem cells into the peripheral circulation during a 4-year follow-up. *International Journal of Cardiology*.

[14] Leone A. M., Rutella S., Bonanno G. (2005). Mobilization of bone marrow-derived stem cells after myocardial infarction and left ventricular function. *European Heart Journal*.

[15] Wyderka R., Wojakowski W., Jadczyk T. (2012). Mobilization of CD34+CXCR4+ stem/progenitor cells and the parameters of left ventricular function and remodeling in 1-year follow-up of patients with acute myocardial infarction. *Mediators of Inflammation*.

[16] Ling L., Shen Y., Wang K. (2012). Worse clinical outcomes in acute myocardial infarction patients with type 2 diabetes mellitus: relevance to impaired endothelial progenitor cells mobilization. *PLOS ONE*.

[17] Shintani S., Murohara T., Ikeda H. (2001). Mobilization of endothelial progenitor cells in patients with acute myocardial infarction. *Circulation*.

[18] Authors/Task Force Members, Gabriel Steg P., James S. K. (2012). ESC Guidelines for the management of acute myocardial infarction in patients presenting with ST-segment elevation. *European Heart Journal*.

[19] O’Gara P. T., Kushner F. G., Ascheim D. D. (2013). 2013 ACCF/AHA guideline for the management of ST-elevation myocardial infarction: a report of the American College of Cardiology Foundation/American Heart Association Task Force on Practice Guidelines. *Circulation*.

[20] Roffi M., Patrono C., Collet J.-P. (2016). 2015 ESC Guidelines for the management of acute coronary syndromes in patients presenting without persistent ST-segment elevation. *European Heart Journal*.

[21] Amsterdam E. A., Wenger N. K., Brindis R. G. (2014). 2014 AHA/ACC guideline for the management of patients with non-ST-Elevation acute coronary syndromes: a report of the American College of Cardiology/American Heart Association Task Force on Practice Guidelines. *Journal of the American College of Cardiology*.

[22] Tahhan A. S., Hammadah M., Raad M. (2018). Progenitor cells and clinical outcomes in patients with acute coronary syndromes. *Circulation Research*.

[23] Massa M., Rosti V., Ferrario M. (2005). Increased circulating hematopoietic and endothelial progenitor cells in the early phase of acute myocardial infarction. *Blood*.

[24] Ceradini D. J., Kulkarni A. R., Callaghan M. J. (2004). Progenitor cell trafficking is regulated by hypoxic gradients through HIF-1 induction of SDF-1. *Nature Medicine*.

[25] Ceradini D. J., Gurtner G. C. (2005). Homing to hypoxia: HIF-1 as a mediator of progenitor cell recruitment to injured tissue. *Trends in Cardiovascular Medicine*.

[26] Cogle C. R., Wise E., Meacham A. M. (2014). Detailed analysis of bone marrow from patients with ischemic heart disease and left ventricular dysfunction: Bm cd34, cd11b, and clonogenic capacity as biomarkers for clinical outcomes. *Circulation Research*.

[27] Kissel C. K., Lehmann R., Assmus B. (2007). Selective functional exhaustion of hematopoietic progenitor cells in the bone marrow of patients with postinfarction heart failure. *Journal of the American College of Cardiology*.

[28] Shantsila E., Watson T., Lip G. Y. H. (2007). Endothelial progenitor cells in cardiovascular disorders. *Journal of the American College of Cardiology*.

[29] Assmus B., Honold J., Schächinger V. (2006). Transcoronary transplantation of progenitor cells after myocardial infarction. *New England Journal of Medicine*.

[30] Tateishi-Yuyama E., Matsubara H., Murohara T. (2002). Therapeutic angiogenesis for patients with limb ischaemia by autologous transplantation of bone-marrow cells: a pilot study and a randomised controlled trial. *The Lancet*.

[31] Bellingan G., Jacono F., Bannard-Smith J. (2022). Safety and efficacy of multipotent adult progenitor cells in acute respiratory distress syndrome (MUST-ARDS): a multicentre, randomised, double-blind, placebo-controlled phase 1/2 trial. *Intensive Care Medicine*.

[32] Pías-Peleteiro J., Campos F., Castillo J., Sobrino T. (2017). Endothelial progenitor cells as a therapeutic option in intracerebral hemorrhage. *Neural Regeneration Research*.

[33] Lee S., Choi E., Cha M.-J., Hwang K.-C. (2015). Cell adhesion and long-term survival of transplanted mesenchymal stem cells: a prerequisite for cell therapy. *Oxidative Medicine and Cellular Longevity*.

[34] Huang W.-H., Chen H.-L., Huang P.-H. (2014). Hypoxic mesenchymal stem cells engraft and ameliorate limb ischaemia in allogeneic recipients. *Cardiovascular Research*.

[35] Fadini G. P., Boscaro E., Albiero M. (2010). The oral dipeptidyl peptidase-4 inhibitor sitagliptin increases circulating endothelial progenitor cells in patients with type 2 diabetes: possible role of stromal-derived factor-1*α*. *Diabetes Care*.

[36] Wang Z., Moran E., Ding L., Cheng R., Xu X., Ma J.-X. (2014). PPAR*α* regulates mobilization and homing of endothelial progenitor cells through the HIF-1*α*/SDF-1 pathway. *Investigative Opthalmology & Visual Science*.

[37] Wang K., Dai X., He J. (2020). Endothelial overexpression of metallothionein prevents diabetes-induced impairment in ischemia angiogenesis through preservation of HIF-1*α*/SDF-1/VEGF signaling in endothelial progenitor cells. *Diabetes*.

