# Supplementary material for: Prognostic Value of Endothelial Progenitor Cells in Acute Myocardial Infarction Patients
Source: Mediators Inflamm. 2023 Sep 28;2023:4450772. doi: 10.1155/2023/4450772 (PMC10613116; doi:10.1155/2023/4450772)
Supplement: Supplementary Materials — Table S1: baseline characteristics among subjects with AMI. Table S2: baseline clinical variables among subjects with AMI (M (Q25 and Q75) or M ± SD). [file 4450772.f1.docx]

**TABLE 1: Baseline characteristics among subjects with AMI**

|  | STEMI  (n=69) | NSTEMI  (n=22) | *t*/*x^2^* | *P* |
| --- | --- | --- | --- | --- |
| Male [n (%)] | 54(78.3) | 15(68.2) | 0.92 | 0.43 |
| Age (years, Mean ± SD)  BMI (kg/m^2^, Mean ± SD) | 64.2±15.8  22.0±2.1 | 71.1±10.0  22.9±2.9 | 1.95  1.71 | 0.06  0.09 |
| APACHEⅡscore (points, Mean ± SD) | 18.2±7.6 | 20.1±7.3 | 1.06 | 0.29 |
| Disease risk factors [n (%)]  Diabetes | 17(24.6) | 6(27.3) | 0.06 | 0.80 |
| Hypertension | 20(29.0) | 8(36.4) | 0.43 | 0.51 |
| Hyperlipidemia | 37(53.6) | 10(45.5) | 0.45 | 0.50 |
| Carotid plaque | 63(91.3) | 18(81.8) | 1.53 | 0.25 |
| Smoking | 56(81.2) | 15(68.2) | 1.64 | 0.24 |
| History of alcoholism | 30(43.5) | 9(40.9) | 0.05 | 0.83 |
| History of coronary atherosclerosis | 13(18.8) | 5(22.7) | 0.16 | 0.76 |
| Family history of cardiovascular disease | 30(43.5) | 10(45.5) | 0.03 | 0.87 |
| Medication history [n (%)]  Aspirin | 17(24.6) | 6(27.3) | 0.06 | 0.80 |
| Clopidogrel hydrogen | 20(29.0) | 8(36.4) | 0.43 | 0.51 |
| Beta blockers | 37(53.6) | 10(45.5) | 0.45 | 0.50 |
| Calcium channel blockers | 63(91.3) | 18(81.8) | 1.53 | 0.25 |
| Killip classifications [n (%)]  Level I-II | 23(33.3) | 6(27.3) | 0.28 | 0.60 |
| Level III-IV | 46(66.7) | 16(72.7) |  |  |

AMI, indicates acute myocardial infarction; STEMI, ST-elevation MI; NSTEMI, non-ST elevation MI; BMI, body mass index.

**TABLE 2: Baseline clinical variables among subjects with AMI [M（Q25, Q75）or M±SD]**

|  | STEMI  (n=69) | NSTEMI  (n=22) | *t*/*U*/*x^2^* | *P* |
| --- | --- | --- | --- | --- |
| cTnI (μg/L) | 54.4 (37.1, 77.2) | 51.5 (41.9, 80.2) | 0.48 | 0.63 |
| NT-proBNP (ng/L)  VIS (points) | 8949.4 (7466.2, 15399.3)  15.2±2.9 | 8932.9 (6069.6, 14047.8)  15.2±3.3 | 0.64  0.03 | 0.52  0.89 |
| GAP (mmHg) | 5.5±1.1 | 5.7±0.9 | 0.63 | 0.53 |
| HCY (umol/L) | 20.6 (14.0, 36.4) | 21.3 (12.1, 33.7) | 720.5 | 0.72 |
| Creatinine (umol/L) | 86.5 (62.5, 103.8) | 76.5 (62.0, 88.5) | 613.0 | 0.18 |
| C-reactive protein (mg/L) | 20.3 (10.3, 44.4) | 35.2 (7.3, 60.1) | 819.5 | 0.58 |
| HbAlc (%) | 6.1±1.7 | 5.7±1.4 | 1.12 | 0.27 |
| CI (L/min/m^2^) | 2.5±0.7 | 2.3±0.6 | 1.19 | 0.24 |
| AB [n (%)]  Three territories | 11(15.9) | 6(27.3) | 2.20 | 0.03 |
| Two territories | 17(24.6) | 2(9.1) |  |  |
| One territory | 41(59.4) | 14(63.6) |  |  |
| CD34+/CD133+ cells (%) | 0.459±0.190 | 0.399±0.152 | 1.35 | 0.18 |
| CD34+/CD133+/KDR+ cells (%) | 0.173±0.065 | 0.176±0.071 | 0.18 | 0.85 |

AMI, indicates acute myocardial infarction; STEMI, ST-elevation MI; NSTEMI, non-ST elevation MI; VIS, vasoactive inotropic score; GAP, central venous-arterial carbon dioxide difference; HCY, homocysteine; CI, cardiac index; AB, atherosclerotic burden.
